# Metabolic Dysregulation in Hepacivirus Infection of Common Marmosets (*Callithrix jacchus*)

**DOI:** 10.1371/journal.pone.0170240

**Published:** 2017-01-13

**Authors:** Cordelia Manickam, Lynn Wachtman, Amanda J. Martinot, Luis D. Giavedoni, R. Keith Reeves

**Affiliations:** 1 Center for Virology and Vaccine Research, Beth Israel Deaconess Medical Center, Boston, Massachusetts, United States of America; 2 New England Primate Research Center, Harvard Medical School, Southborough Campus, Southborough, Massachusetts, United States of America; 3 Southwest National Primate Research Center, Texas Biomedical Research Institute, San Antonio, Texas, United States of America; University of Padua, ITALY

## Abstract

Chronic hepatitis C has been associated with metabolic syndrome that includes insulin resistance, hepatic steatosis and obesity. These metabolic aberrations are risk factors for disease severity and treatment outcome in infected patients. Experimental infection of marmosets with GBV-B serves as a tangible, small animal model for human HCV infection, and while virology and pathology are well described, a full investigation of clinical disease and the metabolic milieu is lacking. In this study six marmosets were infected intravenously with GBV-B and changes in hematologic, serum biochemical and plasma metabolic measures were investigated over the duration of infection. Infected animals exhibited signs of lymphocytopenia, but platelet and RBC counts were generally stable or even increased. Although most animals showed a transient decline in blood glucose, infection resulted in several fold increases in plasma insulin, glucagon and glucagon-like peptide 1 (GLP-1). All infected animals experienced transient weight loss within the first 28 days of infection, but also became hypertriglyceridemic and had up to 10-fold increases in adipocytokines such as resistin and plasminogen activator inhibitor 1 (PAI-1). In liver, moderate to severe cytoplasmic changes associated with steatotic changes was observed microscopically at 168 days post infection. Collectively, these results suggest that GBV-B infection is accompanied by hematologic, biochemical and metabolic abnormalities that could lead to obesity, diabetes, thrombosis and atherosclerosis^,^ even after virus has been cleared. Our findings mirror those found in HCV patients, suggesting that metabolic syndrome could be conserved among hepaciviruses, and both mechanistic and interventional studies for treating HCV-induced metabolic complications could be evaluated in this animal model.

## Introduction

Hepatitis C virus (HCV) causes chronic hepatitis leading to fibrosis, cirrhosis and hepatocellular carcinoma in 80% of infected individuals [1]. About 2.8% of the world’s population is infected with HCV with associated mortality approximating 500,000 deaths per year [2–4]. In addition to liver disease-related mortality, HCV-infected patients are prone to type 2 diabetes and cardiovascular disease [5,6]. A myriad of metabolic aberrations including elevated triglycerides, elevated fasting glucose and abdominal obesity can exacerbate the development of metabolic syndrome, which in turn leads to cardiovascular disease and type 2 diabetes mellitus [7]. Numerous studies have reported the association of HCV and its role in insulin resistance, hepatic steatosis, atherosclerosis and other metabolic aberrations that have been specifically described as HCV-associated dysmetabolic syndrome (HCADS) [8–10]. These metabolic aberrations especially steatosis, have been identified as predictors of poor treatment outcome for interferon-based therapy in chronic HCV infection in the early 2000s [11–14]. In the current era of directly acting antivirals, the impact of metabolic disorders on treatment outcome has not been well studied. A better understanding of the dysmetabolic milieu in HCV-infected patients will be helpful in attaining improved sustained virological response rates followed by successful HCV eradication.

HCV also has a direct role in inducing metabolic dysfunctions. HCV core protein interferes with insulin signaling pathways, thus inducing insulin resistance in the infected patients [15,16]. The expression of HCV non-structural protein 5A (NS5A) in human hepatoma cells lead to upregulated gluconeogenic and lipoegenic gene expression, which in turn favors the development of insulin resistance and metabolic syndrome [16]. In infected hepatocytes, internalized HCV disrupts the host lipid metabolism for its own replication and assembly, leading to hepatic steatosis and non-alcoholic fatty liver disease (NAFLD)/non-alcoholic steatohepatitis (NASH) [9]. Several pathways have been reported to describe HCV mediated lipid dysregulation in a genotypic specific manner. These include hepatic fat accumulation by activation of SREBP-1 and 2, impairment of peroxisome proliferator-activated receptor expression, inhibition of MTP activity and promotion of de-novo lipid synthesis [17–19].

Insulin resistance predates steatosis development, which in turn aggravates steatosis leading to a inflammatory liver microenvironment. This results in activation of cell stress pathways, formation of inflammasome and further hepatocellular injury. Along with liver and pancreas, adipose tissue, acting as an endocrine organ also regulates lipid and glucose metabolism. Dysfunctional adipose tissue is associated with imbalanced production of pro-inflammatory adipokines including adiponectin, monocyte chemoattractant protein-1 (MCP-1), visfatin and others, all contributing to local and systemic metabolic dysregulation [20–24]. A state of chronic, low-level inflammation is associated with the metabolic syndrome, either underlying or exacerbating it, predisposing chronic patients to the risk of developing hepatocellular carcinoma and cardiovascular complications such as atherosclerosis [25–28].

Animal models play an important role in understanding the pathogenesis and immunology of infectious agents, and chimpanzees were formally the primary model for HCV and played a critical role in elucidating the natural history of the disease [29–31]. However, limitations due to ethical and cost reasons have led to a generalized reduction in use of chimpanzees in biomedical research. Marmosets are a promising surrogate nonhuman primate model for HCV due to their high degree of homology with humans immunologically [32]. Most importantly, GB virus-B (GBV-B) belonging to the same family and genus as HCV causes an analogous disease to HCV in new world monkeys, including marmosets [33–37]. In addition to their immunological similarity, marmosets have similar suites of body composition, alterations in glucose and lipid metabolism as observed in humans and other nonhuman primates [38–41]. They are more prone to developing insulin resistance, diabetes mellitus, NAFLD and obesity and are used as models for the same conditions [38–40]. Collectively, the increasing knowledge of marmoset immunology and metabolic pathways, limited size and cost, and availability of cross-reactive reagents makes marmosets an attractive animal model.

## Methods

### Ethics statement and animals

Six common marmosets (*Callithrix jacchus*) were used for this study and were housed in BSL2 biocontainment facilities at the New England Primate Research Center in accordance with the guidelines of the local institutional animal care and use committee, and the Department of Health and Human Services (DHHS) Guide for the Care and Use of Laboratory Animals. The Harvard University IACUC approved all procedures prior to study. All animals were socially housed and enrolled in the NEPRC environmental enrichment program designed to provide mental and sensory stimulation and promote development of behavioral and logical skills using varied stimuli (i.e., foraging devices). Blood draws consisted of no more than 1% of body weight and not more than 3 ml. Post-blood draw analgesics were administered at the discretion of the veterinarian. Animals were fed a commercial new world nonhuman primate diet, which was supplemented with fruits, vegetables, eggs and nuts. Water was available ad libitum. Additional information on the animals used in this study is found in Table 1. Animals were weighed at weekly intervals for the first 4 weeks followed by monthly weight measurements until day 168 post-infection (pi). Sequential blood draws were performed pre- and post- GBV-B inoculation during morning hours for every time point. For all procedures animals were sedated with ketamine (40–50 mg/kg). Animals were sacrificed at 168 days pi with IV administration of an overdose (>>50 mg/kg) of pentobarbital verified by auscultation with a stethoscope. Evident post mortem pathology was recorded by the attending veterinarian and pathologist.

**Table 1:** Pathological lesions in GBV-B infected animals.

Animal	Sex	Age (yrs)	Gross pathology	Steatosis score*
228–2007	Male	7	Enlarged peripheral lymph nodes; splenomegaly; hepatomegaly; kidney pale and mottled	2
16–2007	Male	7	Enlarged peripheral lymph nodes; cardiomegaly; liver enlarged & friable	2
282–2006	Female	8	Enlarged peripheral lymph nodes; splenomegaly; hepatomegaly	2
123–2010	Male	4	No significant findings	1
379–2009	Male	4	Splenomegaly	3
27–2009	Female	5	Splenomegaly; hepatomegaly; kidneys pale, mottled, and cortex pitted	2

* Liver of infected animals were scored for histological changes associated with steatosis at 168 days pi

### Virus inoculum

All animals were inoculated IV with 1.0 x 10^3^ to 4.0 x 10^3^ virus copy equivalents of uncloned GBV-B virus stock as described [42].

### Blood chemistry

Whole blood was also collected at indicated time points and sera were collected at monthly intervals. At pre-infection and monthly time points 0.5 to 1ml (based on body weight of the animal) of whole blood and 1 ml of clotted blood was collected; 0.5ml of blood was collected at day 7, 14 and 21 pi. Blood cell counts were performed by automated analysis (Hemavet HV 1700FS instrument), and biochemical analyses were performed by standard veterinary diagnostics (IDEXX, Grafton MA). The medians and ranges of serum enzymes and analytes of animals at baseline (day 0) are shown in Table 2.

**Table 2:** Serum chemistry baseline/normal values.

Clinical Parameters	Units	Median	Range
ALP	U/L	61	54–73
ALT (SGPT)	U/L	6.5	3–8
AST (SGOT)	U/L	89.5	77–135
CK	U/L	343	187–870
LDH	U/L	308	252–422
GGT	U/L	7.5	3–13
Amylase	U/L	260.5	151–284
Albumin	g/dL	4.05	3.8–4.3
Total protein	g/dL	6.55	6.1–6.7
Globulin	g/dL	2.45	2.1–2.8
BUN	mg/dL	22	19–26
Creatinine	mg/dL	0.4	0.3–0.4
Cholesterol	mg/dL	199	135–223
Glucose	mg/dL	199.5	172–266
Calcium	mg/dL	9.8	9.4–10.2
Phosphorus	mg/dL	3.65	3.0–4.0
TCO2 (Bicarbonate)	mEq/L	21	13–23
Chloride	mEq/L	107.5	103–110
Potassium	mEq/L	3.2	2.8–3.4
Sodium	mEq/L	150.5	146–154
Triglyceride	mg/dL	122.5	77–1017

### Histopathology

Liver samples were processed, stained by haematoxylin and eosin and scored as described [42]. The scores for hepatic steatosis were as follows: None-0, Mild-1, Moderate-2, Severe-3.

### Luminex assay

Marmoset plasma samples were analyzed for glucagon, glucagon like peptide-1 (GLP-1), glucose inhibitory peptide (GIP), insulin, leptin, plasminogen activator inhibitor type -1 (PAI-1), resistin, and visfatin by Luminex [43]. Due to limited availability of samples, plasma samples of only three animals (228–07, 16–07 and 27–09) were analyzed.

### Statistics

Statistical significance of difference was determined by non-parametric Kruskal-Wallis test followed by Dunn’s multiple comparison post-test or paired Students *t* test using GraphPad Prism 6.0 software. Differences between the mean ranks of different time points compared to the mean rank of day 0 were considered significant when the *p* value was less than 0.05. Correlations between metabolic and biochemical factors were analyzed by Spearman correlation test using GraphPad Prism 6.0 software.

## Results

### Clinical presentation of GBV-B infection

**Fig 1:**
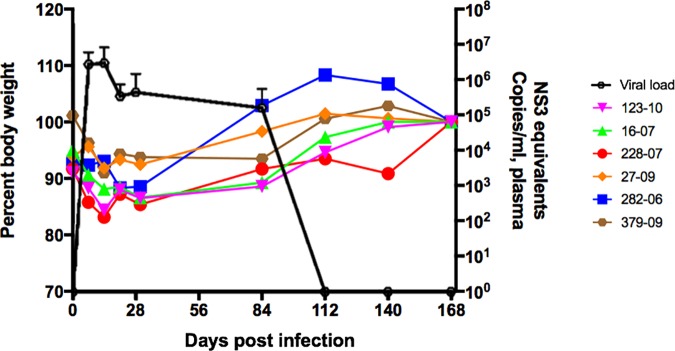
GBV-B viremia and transient weight loss. Kinetics of mean plasma viral load determined by real time PCR and body weight measurements expressed as percent changes compared to baseline values in GBV-B infected marmosets.

Six marmosets were infected with GBV-B and the clinical course of the disease was studied longitudinally over 168 days pi. Viral loads were analyzed in the plasma of infected animals by real time PCR [42]. Post-infection, there was a reduction in percent body weight coinciding approximately with peak mean viremia (Fig 1). Up to 5 to 10% loss in body weight was observed in all animals when compared to baseline values (median = 0.43 kg; range = 0.303–0.496 kg). A return to normal body weight and expected weight gain only occurred after viral clearance.

### Hematological changes in GBV-B infection

**Fig 2:**
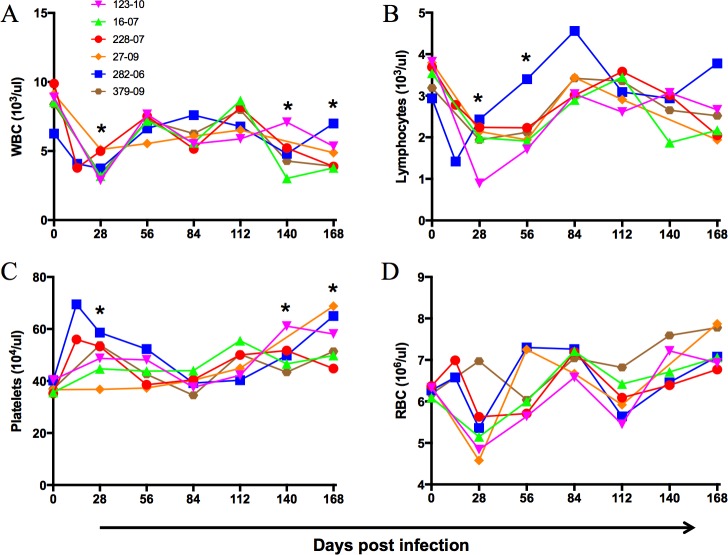
Changes in blood cell counts during GBV-B infection. Changes in cell counts of (A) white blood cells, (B) lymphocytes, (C) platelets and (D) red blood cells of blood from GBV-B infected marmosets. Asterisk indicates significant differences between mean of respective time point and day 0 by Kruskal Wallis test followed by Dunn’s multiple comparison at *p* value < 0.05.

Reduction in the total white blood cell count in all animals was observed at day 28 and at later time points (Fig 2A). Similarly significant loss in lymphocyte count was observed in all animals except 282–06 at days 28 and 56 pi (Fig 2B). Interestingly, platelet counts increased significantly at days 28, 140 and 168 pi (Fig 2C). RBC numbers reduced, although not significantly coinciding with the leukocyte counts at day 28, but not at later time points (Fig 2D). No significant differences were observed in eosinophils, basophils and monocytes (data not shown).

### Biochemical changes indicative of tissue inflammation

**Fig 3:**
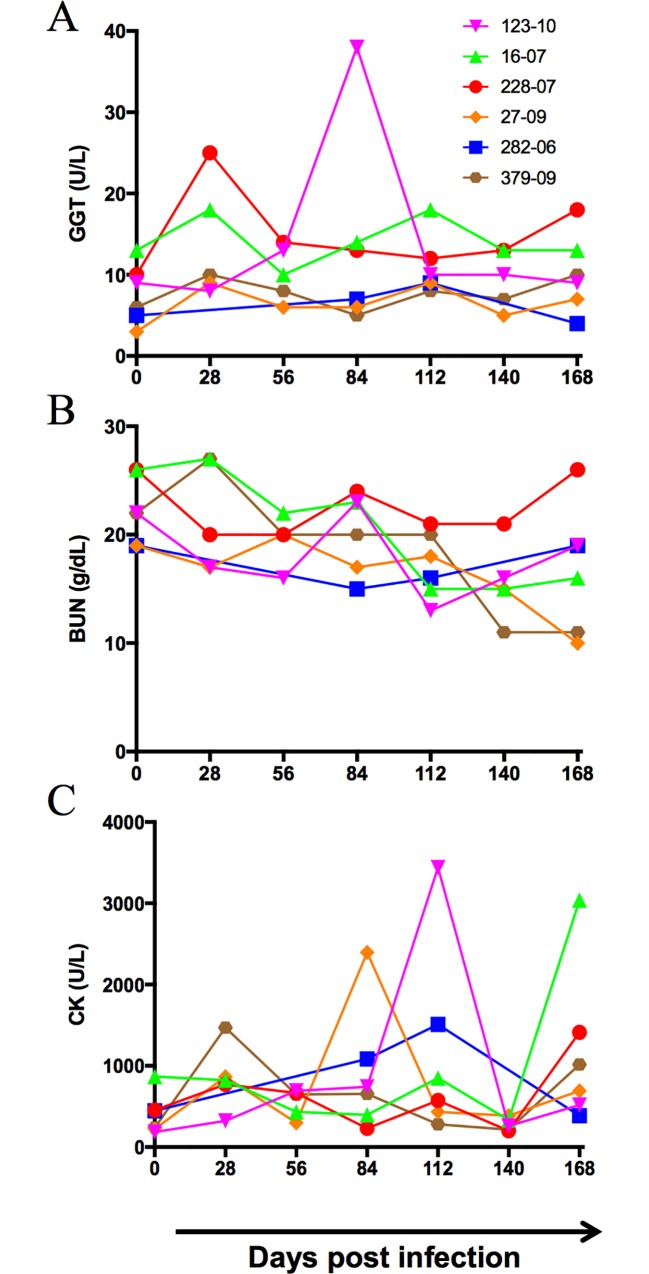
Changes in serum biochemistry. Serum biochemistry for (A) gamma glutamyl transferase (GGT), (B) blood urea nitrogen (BUN), (C) creatine kinase (CK) in GBV-B infected marmosets.

Sera at baseline (day 0, Table 2) and time points’ pi were analyzed for several biochemical parameters. As observed in other reports [44–46], serum enzymes such as alanine aminotransaminase (ALT), aspartate aminotransaminase (AST) and alkaline phosphatase (ALP) indicative of hepatitis were elevated in infected animals although they were not correlated with viral loads [42]. Gamma glutamyl aminotransferase (GGT), another serum enzyme associated with liver damage in marmosets [39], was elevated by at least 1.5 to 2-fold in all infected animals when compared to day 0 values (Fig 3A). Decreases in blood urea nitrogen (BUN) were observed at all time points (Fig 3B). Creatine Kinase (CK) was elevated by more than 2-fold than baseline values in all animals at different time points (Fig 3C) suggesting in addition to liver damage, more generalized activation or cardiovascular damage. No significant changes were observed in levels of albumin, globulin, total proteins and electrolytes such as calcium, phosphorous, chloride, potassium and sodium (data not shown).

### Dysregulated glucose metabolism in GBV-B infection

**Fig 4:**
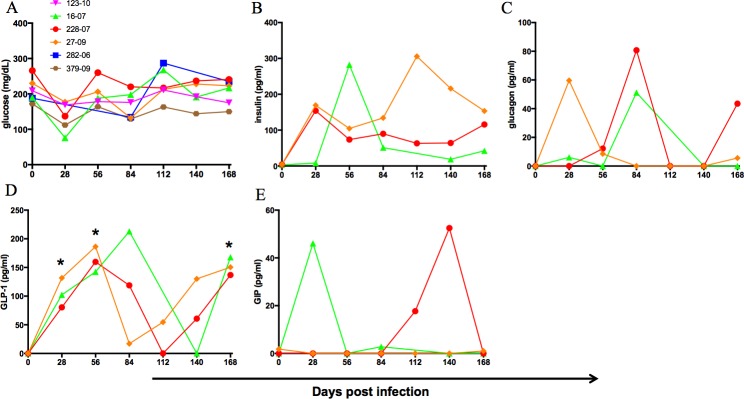
Dysregulated glucose and associated hormones in GBV-B infected animals. Changes in (A) glucose, (B) insulin, (C) glucagon, (D) GLP-1 and (E) GIP in plasma of infected marmosets by luminex assay. Asterisk indicates significant differences between mean of respective time point and day 0 by paired *t* test at *p* value < 0.05.

Serum glucose and plasma hormones involved in glucose metabolism were monitored at monthly time points post infection. A transient but consistent decline in blood glucose was observed by day 28 pi, returning to normal levels by viral clearance (Fig 4A). Based on sample availability, plasma from only three animals—228–07, 16–07 and 27–09 were analyzed for hormones involved in glucose metabolism by Luminex assay. The major pancreatic hormones, insulin and glucagon were increased at multiple time points in all three animals (Fig 4B & 4C). GIP and GLP-1 are incretin hormones produced by the gut. While GLP-1 was elevated at days 28, 56 and 168 at significant levels (Fig 4D) in the three animals, GIP was increased in 228–07 and 16–07 but not in 27–09 (Fig 4E).

### Lipid dysfunctions in infected animals

**Fig 5:**
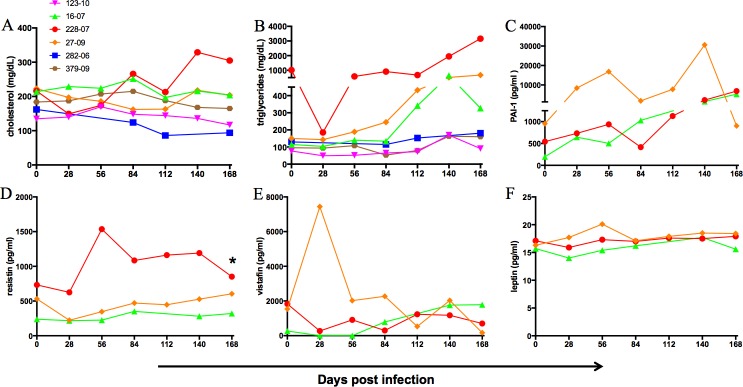
Dyslipidemic profile in GBV-B infected animals. Changes in (A) cholesterol, (B) triglycerides, (C) PAI-1, (D) resistin, (E) visfatin and (F) leptin in plasma of infected marmosets by luminex assay. Asterisk indicates significant differences between mean of respective time point and day 0 by paired *t* test at *p* value < 0.05.

Altered lipid profiles that drive steatosis and insulin resistance have been previously associated with HCV infection [47–49]. In GBV-B-infected marmosets, serum cholesterol levels were generally not variable (Fig 5A), whereas elevated triglycerides were observed at later time points exceeding 400mg/dL in animals 228–07, 16–07 and 27–09 (Fig 5B). Triglyceride concentrations more than 400mg/dL is considered as hypertriglyceridemia in marmosets [38]. Interestingly, the animal 228–07 had high triglyceride levels even at baseline, which further increased by 2 to 3-fold at later time points.

Adipocytokines are cytokines secreted by adipose tissues and are associated with obesity and insulin resistance. PAI-1, visfatin, resistin and leptin are some of the adipocytokines that were analyzed in the plasma of 228–07, 16–07 and 27–09. Increases in PAI-1 as high as 10-fold or more were observed in all three animals at most time points of the study (Fig 5C). Resistin was elevated in animals 228–07 and 16–07 and reached significant levels at day 168 (Fig 5D). Interestingly, resistin levels correlated with triglyceride levels (r = 0.759, p<0.001) in the plasma of infected animals. Visfatin was increased in 27–09 at day 28 pi, and in 16–07 at later time points (Fig 5E). No major changes were observed in leptin levels (Fig 5F).

### Pathological changes towards hepatic steatosis in infected animals

At day 168 pi all infected animals were necropsied and gross pathology was noted. Table 1 lists the various anomalies detected in internal organs of infected marmosets. The most common observations included enlarged liver, spleen and lymph nodes and mottled kidneys.

**Fig 6:**
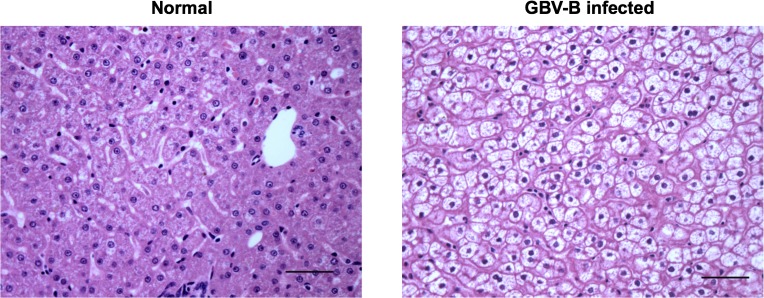
Hepatic steatotic lesions in GBV-B infected animals. Representative micrographs show histopathological changes associated with liver steatosis by H&E (magnification,400x; scale bar 100μm) in healthy and GBV-B infected marmosets.

Previous observations from our lab confirmed the presence of hepatitis and liver fibrosis in GBV-B-infected animals by histopathological evaluation [42]. Further examination for steatosis revealed cytoplasmic vacuoles with peripheral nuclei indicating macrovesicular steatosis in the liver of infected animals at day 168 pi compared to normal animals (Fig 6). Quantitative evaluation of liver histopathology identified varying levels of steatotic changes in all infected animals–ranging from mild steatosis in 123–10 to severe steatosis in 27–09 (Table 1).

## Discussion

Previous studies including our own observations have shown that GBV-B induces acute hepatitis in marmosets and tamarins with elevated serum enzymes such as ALT, isocitrate dehydrogenase and glutamate dehydrogenase [42,44–46]. However, the hematological, biochemical and metabolic changes over the course of GBV-B infection have not been described in detail. In this study, marmosets infected with GBV-B were assessed for changes in both hematological and serum metabolic parameters from onset of infection until after viral clearance.

Many flaviviruses infect hematopoietic cells which, as a general feature of this family of viruses, can lead to neutropenia, bone marrow hypocellularity and abnormal megakaryocyte formation [50]. Thrombocytopenia in combination with leukopenia and hemolysis secondary to HCV infection has been reported [51]. Interestingly, although virus was cleared in most animals by days 28 or 56, recovery of lymphocyte counts was observed much later. However, leukopenia and decreased RBC levels observed in most animals at day 28 could be the result of repeated phlebotomy within the first month of infection. Although thrombocytopenia has been specifically associated with chronic HCV [52–55], platelet counts were not reduced in GBV-B infection. A probable reason for the difference in platelet count between the two hepaciviruses could be the generally acute nature of GBV-B infection which might be insufficient in duration compared to chronic HCV to induce thrombocytopenia.

Dysfunctions of glucose metabolism and insulin resistance are common features in chronic hepatitis due to the major role(s) the liver plays in glucose metabolism. Indeed HCV directly interrupts signaling in the insulin receptor substrate-1 pathway through its core protein [56]. Most infected animals showed evidence of an acute hypoglycemia, but also exhibited elevated levels of insulin, glucagon and GLP-1 (Fig 4). Hyperinsulinemia has been demonstrated in chronic HCV infection [57,58], and both hyperinsulinemia and hyperglucagonemia can be found in cirrhotic patients [59]. GIP is elevated in type 2 diabetes mellitus and in impaired glucose tolerant patients [60,61] while GLP-1 improves insulin sensitivity in mice and humans [62]. However, the changes of glucagon, GLP-1 and GIP hormones in viral hepatitis induced insulin resistance are not completely clear [63,64].

Other than insulin resistance, the metabolic complications most commonly associated with chronic HCV infection include hepatic steatosis and dyslipidemia [65–68]. Patients with hepatic steatosis had significantly increased serum triglycerides than HCV-infected patients without hepatic steatosis [69], and severity of liver injury has been correlated with low cholesterol levels [70–72]. Marmosets infected with a HCV/GBV-B chimera demonstrated pathological changes in liver including lymphocyte infiltration, hepatic edema, cholestasis and ultrastructural changes such as lipid droplets indicative of fatty liver degeneration [73]. In this study, infected animals showed accumulation of lipid in hepatocytes as evidenced by steatosis in liver and additionally hepatomegaly (Table 1), thus recapitulating NAFLD in humans. Further, necrotic hepatocellular structures, inflammatory cell infiltration and mild levels of fibrosis in liver were observed in the same cohort of animals [42] indicating progression of liver injury towards NASH. This data is in accordance with the marmoset model of NAFLD and NASH where elevated serum GGT and triglycerides were suggested as useful biochemical markers of liver dysfunction [39,74].

Obesity and insulin resistance are independent negative predictors for SVR in chronic hepatitis C patients undergoing combination therapy [75]. Adipose tissue derived cytokines or adipocytokines are reported to play an important role in the development of obesity derived insulin resistance [76,77]. Resistin, an adipocytokine was higher in NAFLD patients with moderate/severe liver fibrosis than patients with mild fibrosis [78]. Hyperresistinemia has been reported in Chronic HCV patients [48,79] and IL-8 and resistin levels predicted severe/moderate fibrosis in HCV infected patients [79]. At day 168 pi, significant hyperresistinemia was observed in infected animals. In addition, association between resistin and triglyceride levels indicates that viral hepatitis could drive insulin resistance and lipid dysregulation. PAI-1, another adipocytokine, was elevated in infected animals 2-fold up to 30-fold. PAI-1 levels were associated with triglyceride levels in chronic HCV patients [49]. Elevated PAI-1 levels have been associated with diabetic nephropathy [80,81]. Renal complications such as albuminuria, cryoglobulinemia-induced glomerulonephritis and chronic kidney disease have all been associated with HCV infection [82–84]. Abnormal changes in BUN were observed in some animals and could indicate renal disease. Acute experimental liver damage in cirrhotic rats also induced renal dysfunction with increases in serum creatinine, bilirubin, and BUN levels [85]. Collectively, these data suggest that GBV-B, like many other viral infections, could induce kidney dysfunction, but it is also important to point out that marmosets have a propensity to develop spontaneous benign glomerulonephropathy [86–88]. Thus, further studies will be necessary to determine if kidney disease is a true complication of GBV-B infection. Further, a general state of chronic inflammation is associated with these metabolic aberrations [25–27]. In all GBV-B infected animals, CK was elevated up to 18-fold above baseline. Multiple studies have founded elevated CK and myositis associated with HCV infection [89–92]. This is further supported by the gross pathological observations of hepatomegaly, splenomegaly, kidney lesions and peripheral lymphadenopathy–indicative of an underlying state of inflammation caused by GBV-B infection and similarity to HCV disease.

In summary, despite the relatively low numbers of animals evaluated in this study, our data suggest that GBV-B infection in marmosets could induce significant clinical and metabolic dysregulation as evidenced by hypertriglyceridemia and potential pre-diabetic manifestations similar to disease found in HCV infection of humans. The most notable similarities were signs of systemic inflammation, liver damage, dysfunctional lipid and glucose metabolism, all of which could influence progression of disease and/or response to treatment. Although the measurement of non-fasting plasma glucose is a caveat in this study, several pre-diabetic parameters such as adipocytokine dysregulation in combination with lipid dysregulation indicates a risk of diabetic manifestation. In addition, steatotic changes in liver confirmed triglyceride accumulation leading to fatty liver degeneration. Further, these metabolic aberrations were seen in animals even after virus was completely cleared, which could be indicative of permanent liver damage resulting in progressive dysregulation of metabolic pathways irrespective of virus replication. Therefore, GBV-B infection, regardless of duration, might induce hematologic and metabolic dysfunctions in marmosets similar to those observed in HCV, but the full mechanism of hepacivirus-induced metabolic disease will require further study in additional animal cohorts. Nonetheless, the similarity of disease between the marmoset model and humans could help to better understand the role(s) metabolic dysfunction plays in disease pathogenesis as well as evaluations of these disease complications when considering treatment.
